# Welcome message from the organizers

**DOI:** 10.1186/s42162-020-00114-8

**Published:** 2020-10-28

**Authors:** René Schumann, Roman Rudel, Khoa Nguyen

**Affiliations:** 1 HES-SO Valais Wallis, SILab, Rue de Technpole 3, Sierre, 3960 Switzerland; 2 SUPSI, Trevano - Blocco B, Via Trevano, Canobbio, 6952 Switzerland

Dear readers,

The recent environmental and societal developments, in particular, the climate change happening more and more visible to larger parts of the society and also the COVID-19 pandemic has shown that changes in society, economy and all other energy-usage areas of our societies are needed but also possible. At the same time, we have also seen the new rise of AI / Informatics, which is now considered the “new hope” as a technological enabler for numberless solutions, either easing the required transition, making our lives safer or more convenient. Energy Informatics is therefore needed more than ever, to create an environment for researchers and practitioners from different areas of energy systems as well as information technology to present new results and discuss innovative approaches and solutions. We are happy to present the proceedings of DACH+ Energy Informatics 2020, which is held in Sierre, Switzerland in October 2020.

Despite interruptions from the current pandemic, the DACH+ Energy Informatics series has continued to establish itself as the premier European conference in the field. This year, we received 42 submissions. The submissions came from 7 countries, which cover a wide range of topics such as smart grid, renewable energy, blockchain, electrical vehicle, etc. The Technical Programming Committee gave feedback on the submissions in the form of more than 120 reviews. For the third year of open-access proceedings in the Springer Journal of Energy Informatics, 15 full and short research contributions and 16 poster abstracts were accepted, resulting in an acceptance rate of 36%.

This year’s conference is organized and hosted by the University of Applied Sciences in Western Switzerland - HES-SO Valais-Wallis. Publication funding was provided by the Swiss Federal Office of Energy.

Sincerely,

René Schumann General Chair

Roman Rudel Technical Program Chair

Khoa Nguyen Publication Chair

## Data Availability

Not applicable.

